# Evaluating Targeted Recruitment Interventions and Outcomes to Measure Diversity in Emergency Medicine: A Scoping Review

**DOI:** 10.7759/cureus.105773

**Published:** 2026-03-24

**Authors:** Sherell Hicks, Erin F Shufflebarger, Zachary Pacheco, James Gilbreath, Logan Wilson, Christopher Reisig, Teresa M Chan

**Affiliations:** 1 Emergency Medicine, University of Alabama at Birmingham Heersink School of Medicine, Birmingham, USA; 2 Research, University of Alabama at Birmingham Heersink School of Medicine, Birmingham, USA; 3 Emergency Medicine, Vanderbilt University Medical Center, Nashville, USA; 4 Emergency Medicine, Weill Cornell Medicine, New York, USA; 5 Emergency Medicine, Toronto Metropolitan University, William Osler Health Systems, Brampton, CAN

**Keywords:** diversity equity and inclusion (dei), recruitment strategies, residents and faculty, special interest in emergency medicine, underrepresented gender and racial minority

## Abstract

As the US national population continues to diversify, there have been access and engagement efforts within emergency medicine (EM) to help create a workforce that is more representative of the patients we treat. While the importance of diversity is well recognized, there has not been an established way to measure the effectiveness of diversity interventions beyond demographics. This scoping review aims to identify targeted interventions to recruit underrepresented groups in EM and to determine appropriate outcomes to measure diversity in any field that can be applied to EM.

We conducted a literature search in March 2023 using the PubMed, Scopus, and Web of Science databases for articles published from 2008 to the present. Abstracts were independently reviewed by two reviewers to identify potentially relevant articles, and the full text of articles meeting the inclusion criteria was subsequently reviewed. Selected articles were organized into two categories: interventions within EM or measurable outcomes. The reported outcomes were then grouped into themes. The literature search identified 4,696 total abstracts for the review, of which 53 were for interventions in EM and 4,643 for outcomes. Of the 157 abstracts selected for full-text review, 69 articles were selected for inclusion. Fourteen articles identified strategies to recruit underrepresented groups into EM at all levels through pipeline programs, recruitment initiatives, transparent compensation/promotion, and faculty development programs. A total of 55 articles provided measurable outcomes, with 74.5% focused on gender, 7.3% on underrepresented minorities in medicine, and 18.2% focused on both. The derived outcomes from the 55 articles were organized into the following themes in order of most commonly mentioned: promotion/rank (35 out of 55, 63.6%), leadership positions (32 out of 55, 58.2%), H-indices (21 out of 55, 38.2%), number of publications (18 out of 55, 32.7%), and compensation (14 out of 55, 25.5%). Additional outcomes to consider included authorship (first vs. last), national speaking opportunities, national awards, and grant funding.

Overall, this scoping review discovered diversity initiatives within EM and appropriate outcomes to measure diversity. The next step is to use these outcomes to determine whether our efforts are having the intended effect of promoting equity and inclusion in EM.

## Introduction and background

The US population continues to diversify, with predictions of continued growth in the foreseeable future [1]. Despite this advancement, the diversity of the medical workforce continues to lag behind the positive national growth trend. This discrepancy is particularly important in emergency medicine (EM), as the emergency department (ED) serves as the healthcare system's safety net, with physicians frequently addressing health disparities and treating patients regardless of social status. In 2008, the Council of Emergency Medicine Residency Directors (CORD) issued a call to action urging residency programs to become more representative of the patient population presenting to the ED, recommending interventions to increase diversity within residency programs, which will lead to a more diverse workforce in EM [2]. It has been well-established that diversity within the medical workforce improves health outcomes, fosters higher quality and culturally sensitive care, provides financial benefits within healthcare, and leads to decreased health disparities [3,4]. While the positive impact of diversity has been proven, a way to measure the effectiveness of interventions to improve diversity, equity, and inclusion (DEI) beyond demographics has not been.

In order to delineate what literature exists regarding this topic, we determined that a scoping review would be the best way to answer the following two questions: (1) What interventions are being implemented to recruit underrepresented groups to EM after the call to action from CORD? (2) What measurable outcomes in any field are being used to assess if equity and inclusion are being achieved? Following from these two questions, the objectives of our scoping review were to identify targeted interventions to recruit underrepresented groups in EM and to determine appropriate outcomes to measure DEI in any field that can be applied to EM.

## Review

Methods

Search Strategy

We conducted a scoping review in accordance with the guidelines set by Peters et al. [5], an update of the PRISMA-ScR guidelines [6]. Due to the nature of the research question, we built and conducted two searches in March 2023. With the consultation of an academic research librarian (author JNG), we created the first search related to interventions for recruitment of underrepresented in medicine (URiM) physicians in EM. We used the URiM definition included in the Council of Residency Directors (CORD) recommendations and established by the Association of American Medical Colleges, which defines URiM as “racial and ethnic populations that are underrepresented in the medical profession relative to their numbers in the general population” [2]. A comprehensive search was written for PubMed and translated for Scopus and Web of Science. The search included the terms "under-represented in medicine," various race and ethnicity terms, "emergency medicine," and terms related to interventions, outcomes, strategies, and initiatives. For the full search string, see Appendix A. Since we were primarily interested in the US context, we restricted the results to English-language articles as designated by Web of Science and Scopus. We also restricted the results from 2008 to the present, as the CORD recommendations for promoting diversity were released in 2008 [2].

For the second search, we created a search on outcomes and measurements of DEI initiatives in medical education with consultation from the same academic research librarian. A comprehensive search was written for PubMed and translated for Scopus and Web of Science. The search included terms related to DEI, promotion and retention of physicians, and terms for physicians. The search used the same restrictions as above. For the full search string, see Appendix B. 

Screening and Selection

Search results were uploaded to Covidence (Veritas Health Innovation, Melbourne, AUS), a web-based collaboration platform that streamlines the production of systematic and other literature reviews [7], for deduplication and study selection. Our team screened abstracts in Covidence using the inclusion and exclusion criteria specified in Table 1. Two members of our team independently reviewed all the abstracts, with full articles being selected for review if both members agreed on inclusion. A third reviewer served as a tiebreaker for the selection of articles for full-text review. During full article review, two reviewers determined if the article met the final inclusion criteria. Any disagreements were resolved by consensus between the reviewers.

**Table 1:** Inclusion and exclusion criteria for the scoping review EM: Emergency medicine, URiM: Underrepresented in medicine

Parameters	Inclusion criteria	Exclusion criteria
Targeted interventions to recruit URiM groups to Emergency Medicine	Must be within EM	Interventions in another field or medical specialty
	Includes specific interventions or initiatives to recruit URiM groups	Unavailable in English
	Available in English	-
Measurable outcomes for diversity	Any field or medical specialty	No measurable outcome for diversity beyond demographics
	Identifies at least one measurable outcome for diversity (leadership positions, publications, promotion/rank, etc.)	Unavailable in English
	Available in English	-

Data Synthesis and Statistical Analysis

We created a data extraction form to organize the content from selected articles into groups and themes. Included articles were organized into two categories: targeted interventions to recruit URiM groups within EM and measurable outcomes for DEI. We divided these categories into themes using inductive thematic analysis. The themes derived from the full article review included targeted interventions involving pipeline programs, recruitment initiatives, transparency in promotion and compensation, and faculty development programs. In regard to measurable outcomes, we grouped the articles focusing on URiM, gender, or both. The specific measurable outcomes extracted from the included articles were summarized and divided into the following major categories: promotion/rank, leadership positions, H-indices, number of publications, and compensation. No meta-analysis was conducted due to the heterogeneity of study designs and outcomes. Data were summarized and reported using descriptive statistics calculated as percentages of articles discussing an intervention and frequency counts of a reported outcome from the data extraction form. A critical appraisal of the individual articles was not done, as that is beyond the focus of this scoping review [5,6]. 

Results

Study Selection

**Figure 1:**
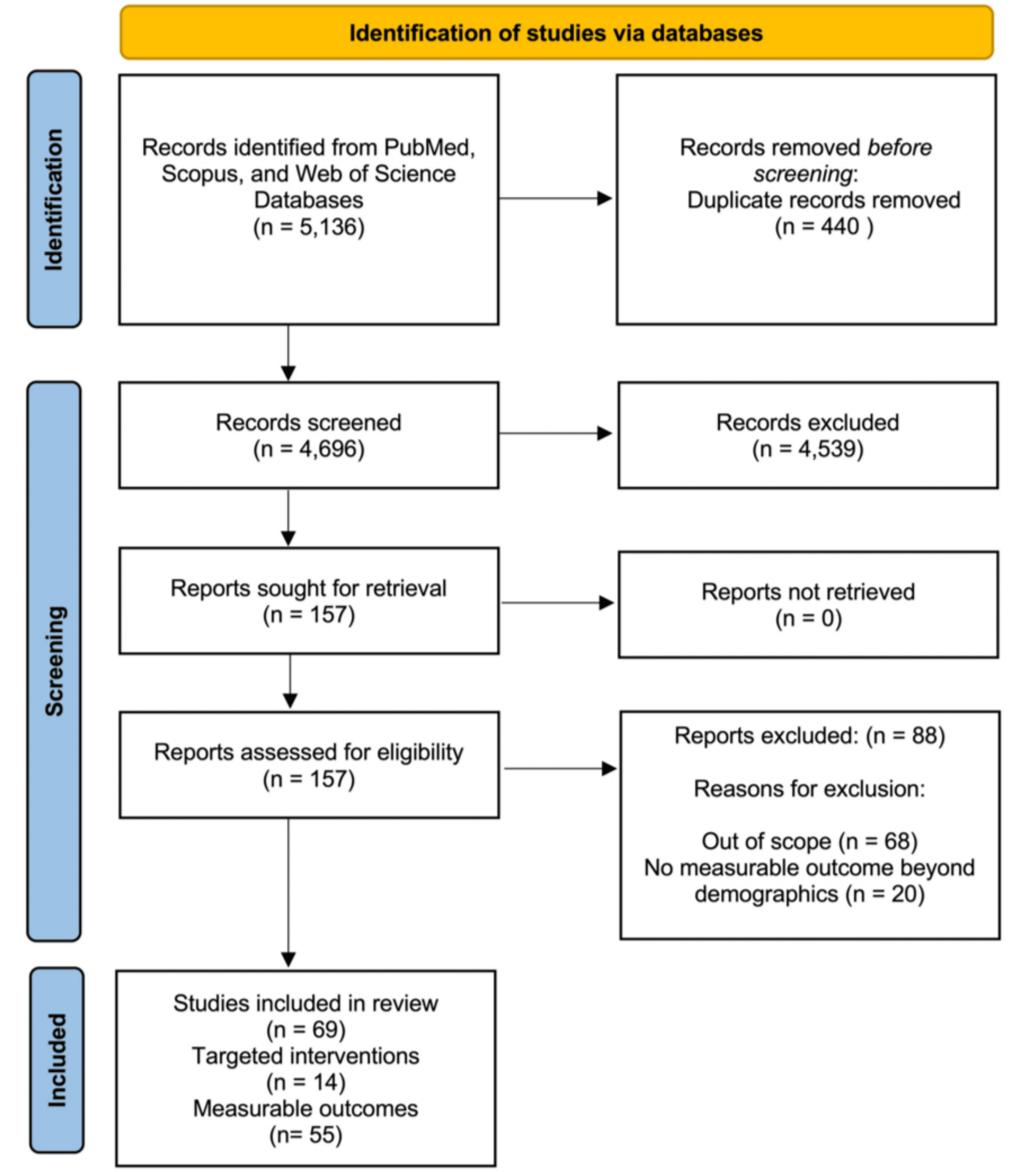
PRISMA flow diagram for scoping review PRISMA: Preferred Reporting Items for Systematic reviews and Meta-Analyses [8]

The search for interventions to recruit URiM groups to EM resulted in 63 articles, and the search for outcomes to measure diversity resulted in 5,073 articles, for a combined total of 5,136 articles. Covidence identified 440 duplicates, resulting in 4,696 abstracts for screening. Of these abstracts, 157 were selected for potential inclusion and qualified for full article review, which identified 69 articles for final inclusion. Of the 69 articles, 14 articles focused on specific interventions to recruit URiM groups to EM, and 55 articles focused on measurable outcomes for diversity in any field (Figure 1) [8]. 

Targeted Interventions 

Fourteen articles identified specific interventions to recruit URiM groups to EM (Table 2). Regarding the population of interest for these interventions, seven out of 14 articles (50%) focused on medical students, five out of 14 (36%) focused on faculty, one out of 14 (7%) focused on both faculty and medical students, and another one out of 14 (7%) focused on students at all levels. The derived themes of the EM-specific interventions included pipeline programs, recruitment initiatives, transparency in promotion and compensation, and faculty development programs. For pipeline programs, three articles discussed implementing programs to garner more interest in EM from offering visiting electives focused on healthcare disparities, establishing a partnership with a historically Black college without an EM residency, and engaging students early on to foster interest in EM through workshops to prepare students for medical school [9-11].

**Table 2:** Extracted data for targeted interventions in EM to recruit underrepresented groups URiM: Underrepresented in medicine, EM: Emergency medicine

Article	Population	Interventions
Boatright et al. (2016) [9]	Medical students	Pipeline programs (visiting elective rotation scholarship and health disparities externship), holistic review
Boatright et al. (2018) [12]	Medical students, Faculty	Medical students-visiting rotations/stipends for URiM, engagement with minority medical student organizations, dedicated second look weekends for URiM applicants, holistic review of residency applications; faculty-initiatives for recruitment and retention, mentorship, faculty development, and promotion
Gallegos et al. (2022) [13]	Medical students	Intentional recruitment strategies, even at the pre-med level; screening/interview process to mitigate bias; holistic review; dedicated second look visits
Garrick et al. (2019) [14]	Medical students	Recruitment initiatives such as a recruitment dinner, holistic review, dedicated committee to review URiM applicants
Tunson et al. (2016) [15]	Medical students	Recruitment initiatives such as an away rotation scholarship and a dedicated second look
Winfield et al. (2020) [16]	Medical students	Recruitment initiatives such as away rotation scholarship, intentional social events while rotating, and involvement in minority student organizations
Goines et al. (2020) [10]	Medical students	Pipeline program (partnership between Emory Department of Emergency Medicine and Morehouse School of Medicine); recruitment initiatives, including paid elective; funded second look visit; mentorship
Heron et al. (2009) [2]	Medical students	Recruitment initiatives, holistic review, and outreach programs to engage students in EM; highlight residency curriculum centered on cultural competency
Davenport et al. (2022) [17]	Faculty	Initiatives for recruitment and retention, faculty development programs, creation of leadership roles at an institutional level, and mentorship
Love et al. (2022) [18]	Women faculty	Mentorship, women’s professional development groups to support advancement/promotion
Madsen et al. (2022) [19]	Faculty	Structured and transparent promotion process, mentorship, and salary transparency
Oh et al. (2021) [20]	Faculty	Transparency in the promotion process, mentorship, and faculty development initiatives
Parsons et al. (2022) [11]	Students at all levels (elementary to medical students)	Pipeline, pathway, and outreach programs-exposure to healthcare early on, workshops to help with Medical College Admission Test (MCAT)/medical school application process, visiting rotations, post- baccalaureate programs
Pierce et al. (2019) [21]	Faculty	Mentorship, faculty development initiatives

For student recruitment initiatives, eight studies discussed funding scholarships to support URiM students during their visiting rotations, hosting a separate second look event for URiM students, getting involved in national URiM student organizations, and utilizing holistic review in the residency application screening process [2,9,10,12-16]. Specifically looking at faculty recruitment initiatives, there was a focus on not only recruitment but also retention of URiM faculty, with two articles detailing how to create a culture of inclusivity at the departmental and institutional levels [12,17]. Transparency in promotion and compensation was highlighted in five articles with initiatives focusing on receiving credit for work not traditionally recognized as eligible for promotion; creating leadership roles within the department and institution for equity and inclusion efforts; and eliminating bias in the promotion and tenure process, as well as the hiring process with equitable compensation [12,17-20]. Faculty development programs centered on providing opportunities for mentorship and sponsorship to enhance academic productivity as well as the development of professional skills, for example, in leadership and grant/manuscript writing [12,17-21].

The overall trend for specific interventions implemented by residency programs and academic institutions within EM to recruit underrepresented groups to medicine focuses on engaging learners early on through pipeline programs to increase exposure. Once medical students begin to consider residency options, the most common recruitment initiative has been financial support for away rotations as well as establishing a presence within national URiM student organizations. Individual programs rely on their local connections to create more robust URiM initiatives by partnering with neighboring historically black colleges. At the faculty level, less emphasis is on recruitment, with the focus centering on retaining faculty through building an inclusive culture, recognizing URiM efforts towards promotion, and providing mentorship for professional development.

Measurable Outcomes for Diversity 

A total of 55 articles provided measurable outcomes for diversity beyond demographics (Table 3). Of the 55 included articles, 41 (75%) focused on gender, four (7%) on URiM, and 10 (18%) focused on both. The most common population analyzed was faculty in 51 out of 55 studies (92%). Most of the articles (46, 84%) were related to the medical field with a focus on surgical subspecialties (24, 44%). The majority of articles focused on academic advancement, with 35 (63.6%) discussing promotion/rank and 32 (58.2%) discussing leadership positions. Another major theme centered on research, with 21 articles (38.2%) utilizing H-indices and 18 (32.7%) using the number of publications, and only a small subset (two, 3.6%) focused on grant funding and order of authorship. Additionally, financial outcomes were mentioned, with compensation being addressed in 14 out of 55 articles (25.5%). Only a few studies (four, 5.5%) included national presence, specifically analyzing national speaking opportunities and national awards. 

**Table 3:** Extracted data for outcomes to measure diversity URiM: Underrepresented in medicine

Article	Gender/URiM/Both	Measurable outcomes
Abelson et al. (2018) [22]	URiM	Promotion/rank, retention
Aberg et al. (2017) [23]	Both	Promotion/rank, compensation
Acosta et al. (2020) [24]	Both	Promotion/rank, leadership positions
Agaronnik et al. (2022) [25]	Gender	Promotion/rank, leadership positions, publication numbers/counts, H-indices, first author/senior author, time to publication, high-impact journals
Ahmadi et al. (2018) [26]	Gender	Promotion/rank, leadership positions, publication numbers/counts, H-indices
Arya et al. (2022) [27]	Gender	Promotion/rank, leadership positions
Berghoff et al. (2021) [28]	Gender	Leadership positions, H-indices, first and last authorship, invited speaker roles at national conferences, board members of societies
Berry et al. (2020) [29]	Both	Promotion/rank, leadership positions, NIH grant funding
Brisbin et al. (2023) [30]	Gender	Promotion/rank, leadership positions, publication numbers/counts, H-indices
Butkus et al. (2018) [31]	Gender	Leadership positions, compensation
Calderwood et al. (2021) [32]	Gender	National awards
Chauvin et al. (2019) [33]	Gender	Publication numbers/counts, H-indices
Chen et al. (2020) [34]	Gender	Promotion/rank, leadership positions, publication numbers/counts, H-indices
Chou et al. (2023) [35]	Both	Promotion/rank, H-indices
de Rosner-van Rosmalen et al. (2022) [36]	Gender	Leadership positions
Diatta et al. (2022) [37]	URiM	Promotion/rank, leadership positions, publication numbers/counts, H-indices
Do et al. (2020) [38]	Both	Promotion/rank, leadership positions, compensation, H-indices
Donaldson et al. (2021) [39]	Gender	Promotion/rank, leadership positions, H-indices
Ehrlich et al. (2021) [40]	Gender	Leadership positions
Esslinger et al. (2020) [41]	Gender	Promotion/rank, leadership positions, publication numbers/counts, H-indices
Fang et al. (2021) [42]	Gender	National awards
Freund et al. (2016) [43]	Gender	Compensation
Gerull et al. (2021) [44]	Gender	National awards
Gerull et al. (2020) [45]	Gender	Annual meeting speaking opportunities
Ha et al. (2021) [46]	Gender	Promotion/rank, H-indices
Han et al. (2017) [47]	Gender	Leadership positions
Henderson et al. (2014) [48]	Gender	Promotion/rank, compensation, H-indices
Horowitz et al. (2021) [49]	Gender	Leadership positions, compensation, publication numbers/counts
Horowitz et al. (2021) [50]	URiM	Promotion/rank, leadership positions, compensation, publication numbers/counts
Hunter et al. (2021) [51]	Gender	Promotion/rank, leadership positions
Hunter et al. (2022) [52]	Gender	Promotion/rank, leadership positions
Hwang et al. (2017) [53]	Both	Promotion/rank
Jagsi et al. (2013) [54]	Gender	Compensation
Kearns et al. (2020) [55]	Gender	Promotion/rank, compensation
Kearns et al. (2022) [56]	Gender	Promotion/rank, leadership positions, H-indices
Krise et al. (2020) [57]	Gender	Leadership positions, publication numbers/counts
Lin et al. (2022) [58]	Gender	NIH grant funding
Linden et al. (2022) [59]	Both	Promotion/rank, leadership positions
Loder et al. (2023) [60]	Gender	Promotion/rank, compensation
Madsen et al. (2017) [19]	Both	Promotion/rank, leadership positions, compensation
Moghimi et al. (2019) [61]	Gender	Promotion/rank, leadership positions, publication numbers/counts, H-indices
Patel et al. (2022) [62]	Gender	Leadership positions, publication numbers/counts, H-indices
Purdy et al. (2021) [63]	Gender	Publication numbers/counts, H-indices, first and last authorship, grant funding
Qamar et al. (2018) [64]	Gender	Promotion/rank, H-indices
Radford et al. (2022) [65]	Gender	Promotion/rank, H-indices
Sing et al. (2017) [66]	Gender	Publication numbers/counts, first and last authorship
Smith et al. (2019) [67]	Gender	Promotion/rank, leadership positions
Smith et al. (2020) [68]	URiM	Promotion/rank, leadership positions
Smith and Jacobson (2016) [69]	Both	Compensation
Smith et al. (2017) [70]	Gender	Compensation
Talbott et al. (2023) [71]	Gender	Promotion/rank, leadership positions, publication numbers/counts
Waseem et al. (2019) [72]	Gender	Promotion/rank, publication numbers/counts, H-indices
Winkelman et al. (2020) [73]	Gender	Promotion/rank, leadership positions, compensation, publication numbers/counts, NIH funding
Wooding et al. (2020) [74]	Both	Promotion/rank, leadership positions
Wu et al. (2019) [75]	Gender	Promotion/rank, leadership positions, publication numbers/counts, H-indices

Discussion

This scoping review reveals that multiple targeted interventions to recruit underrepresented groups to EM have been implemented at all levels, from undergraduate students to faculty, since the call to action from CORD. However, these interventions are not universal and are residency program, department, or institution dependent.

Barriers to DEI interventions

Widespread acceptance and development of initiatives for DEI efforts have not occurred for a few reasons. Many articles report multiple challenges that prevent DEI from being a top priority, including a lack of institutional support, difficulty mitigating bias, the minority tax of expecting URiM groups to solely support DEI work voluntarily, and time-intensive efforts not consistently yielding intended results [3,12,17]. Some articles mentioned there was previously a paucity of actionable ways to achieve diversity, but the CORD recommendations specifically for EM residency programs alleviated this barrier, supporting the concept that institutional buy-in is essential to enact these interventions successfully [2,3]. The Accreditation Council of Graduate Medical Education recognizes the importance of creating a diverse workforce through its five-point framework, which emphasizes how essential leadership involvement is to promoting a diverse culture of inclusivity [3]. Changing the culture involves starting at the residency application and selection process through holistic review and includes dedicated training for faculty in hopes of reducing bias as well as transforming and promoting an inclusive culture within the department [3,17]. These interventions highlight the importance of not only recruitment efforts to attract URiM residents and faculty but also starting from within the department to ensure these individuals are supported once they arrive. 

Outcomes and Implications

Extracting measurable outcomes is crucial to assessing if interventions to improve diversity are successful, as these results attest to the ultimate goal of equity and inclusion being achieved. The majority of the articles highlighted the importance of analyzing beyond demographics to ensure that underrepresented groups are not only increasingly entering the medical field and helping to create a more diverse workforce, but also resulting in more equitable and inclusive opportunities. The focus of the majority of the articles on gender is likely due to gender encompassing a larger subset compared to URiM. Although some areas are improving, the overwhelming majority of reviewed articles show disparities through either gender or URiM. 

Career Advancement

Traditionally, DEI efforts were not recognized for promotion and fell solely on URiM faculty, hence the minority tax and less time for other academic endeavors, contributing to less diverse representation at higher levels of academia [12,17]. Similar trends were seen with gender as increasing numbers of women are present within medical school, residency, and subsequently the workforce, but inequities still exist within leadership positions and promotion. Potential reasons are believed to be systemic, as there is no longer a pipeline discrepancy with women comprising half of the medical school graduates consistently for years [22]. Furthermore, women face challenges that include traditional family obligations, work-life balance, and discrimination/bias in the workplace that can limit opportunities for advancement [22,23]. 

Scholarly Activity

Additionally, disparities were present pertaining to scholarly activity in regard to authorship, grants, and publications. Notably, the lack of female mentorship specifically in research-focused surgical subspecialties likely contributes to a slower academic start that can impede research progression to senior authorship, leading to delayed advancement and impacting research productivity as well as promotion [24]. This concept further emphasizes that diversity begets diversity and can have a long-lasting impact on career trajectories [9]. 

Compensation

Lastly, financial discrepancies were found to exist amongst gender and underrepresented groups even when adjusting for confounding factors, with the largest pay gap between White men and Black women physicians [23]. Potential etiologies include less negotiation and self-advocacy during the hiring process, emphasizing the need for mentorship to employ strong negotiation skills upfront to advocate for equitable pay [22,23]. Although some outcomes showed no significant difference between gender and URiM within certain specialties, the majority identified disparities that need to be addressed. The inequities that exist are multifactorial and present in various medical subspecialties, but they show how much work needs to be done to continue to strive for equity and inclusion. 

Limitations

Our searches utilized PubMed, Scopus, and Web of Science databases; therefore, it is possible that relevant articles published in another database that met our inclusion criteria could have been missed. However, based on how we conducted our search, it is very unlikely that any pertinent articles were inappropriately excluded. Furthermore, the time constraints on our search of only reviewing articles since the call to action by CORD in 2008 could have excluded references that started EM-specific interventions before 2008. It would be unlikely that relevant articles were missed, as the call to action was made due to the paucity of DEI efforts in EM. Additionally, some studies may have potentially misclassified what is defined as URiM. Our search only included studies that were in English, which possibly limited outcomes from international studies. Lastly, most of the included articles are related to healthcare, and we could have missed additional outcomes for consideration in non-medical-related fields based on the databases used. 

## Conclusions

This scoping review identified EM-specific interventions that have been implemented to recruit URiM groups, as well as measurable outcomes from other fields to assess the success of DEI efforts. Next steps include applying these outcomes to EM to determine if the interventions are having their intended effect of improving equity and inclusion within the EM workforce. Although increased DEI recommendations and efforts have occurred and the benefits of a diverse healthcare workforce have been proven, it will be more challenging going forward to assess the impact, given the recent shift of prior legislation that encourages less focus on DEI initiatives. However, the importance of DEI efforts is undisputed as they positively impact patient care.

## Appendices

Appendix A

Search terms for targeted interventions for URiM, used in PubMed, Scopus, and Web of Science, limiting results to English-language articles related to the US from 2008 to the present, yielded 63 articles (Table 4).

**Table 4:** Search for targeted interventions for URiM (total n after deletion of duplications = 63) URiM: Underrepresented in medicine, MeSH: Medical subject headings

Database	MeSH terms	No. of results
PubMed	1. ”Under-represented in medicine"[tiab] OR "Under Represented in Medicine"[tiab] OR "Underrepresented in Medicine"[tiab] OR URiM[tiab] OR URM[tiab] OR "underrepresented medicine"[Title/Abstract:~4] OR (("medical education"[tiab] OR "graduate medical education"[tiab] OR "medical school"[tiab]) AND (ethnic*[tiab] OR Black[tiab] OR "african american"[tiab] OR arab*[tiab] OR "asian american"[tiab] OR "native hawaiian"[tiab] OR "pacific islander"[tiab] OR AAPI[tiab] OR hispanic[tiab] OR latin*[tiab] OR indigenous[tiab] OR "native american"[tiab] OR Jewish[tiab] OR roma[tiab] OR "minority group*"[tiab] OR "ethnic and racial minorit*"[tiab])) OR ("Education, Medical"[Mesh] AND ("Ethnicity"[Mesh] OR "Black or African American"[Mesh] OR "Arabs"[Mesh] OR "Asian American Native Hawaiian and Pacific Islander"[Mesh] OR "Hispanic or Latino"[Mesh] OR "Indigenous Peoples"[Mesh] OR "Jews"[Mesh] OR "Roma"[Mesh] OR "Minority Groups"[Mesh] OR "Ethnic and Racial Minorities”[Mesh])); 2. outcome[tiab] OR intervention[tiab] OR initiative[tiab] OR strateg*[tiab]; 3. "emergency medicine"[tiab] OR EM[tiab] OR "Emergency Medicine”[Mesh]; 4. #1 AND #2 AND #3; 5. Limit 2008 to Present	52
Scopus	TITLE-ABS("Under-represented in medicine") OR TITLE-ABS("Under Represented in Medicine") OR TITLE-ABS("Underrepresented in Medicine") OR TITLE-ABS(URiM) OR TITLE-ABS(URM) OR ((TITLE-ABS("medical education") OR TITLE-ABS("graduate medical education") OR TITLE-ABS("medical school")) AND (TITLE-ABS(ethnic*) OR TITLE-ABS(black) OR TITLE-ABS("african american") OR TITLE-ABS(arab*) OR TITLE-ABS("asian american") OR TITLE-ABS("native hawaiian") OR TITLE-ABS("pacific islander") OR TITLE-ABS(AAPI) OR TITLE-ABS(hispanic) OR TITLE-ABS(latin*) OR TITLE-ABS(indigenous) OR TITLE-ABS("native american") OR TITLE-ABS(jewish) OR TITLE-ABS(roma) OR TITLE-ABS("minority group*") OR TITLE-ABS("ethnic and racial minorit*”))) AND (TITLE-ABS(outcome) OR TITLE-ABS(intervention) OR TITLE-ABS(initiative) OR TITLE-ABS(strateg*)) AND (TITLE-ABS(“emergency medicine") OR TITLE-ABS(EM))	19 (10 duplicates were removed, resulting in nine remaining articles)
Web of Science	((TI="Under-represented in medicine" OR AB="Under-represented in medicine") OR (TI="Under Represented in Medicine" OR AB="Under Represented in Medicine") OR (TI="Underrepresented in Medicine" OR AB="Underrepresented in Medicine") OR (TI=URiM OR AB=URiM) OR (TI=URM OR AB=URM) OR (((TI="medical education" OR AB="medical education") OR (TI="graduate medical education" OR AB="graduate medical education") OR (TI="medical school" OR AB="medical school")) AND ((TI=ethnic* OR AB=ethnic*) OR (TI=black OR AB=black) OR (TI="african american" OR AB="african american") OR (TI=arab* OR AB=arab*) OR (TI="asian american" OR AB="asian american") OR (TI="native hawaiian" OR AB="native hawaiian") OR (TI="pacific islander" OR AB="pacific islander") OR (TI=AAPI OR AB=AAPI) OR (TI=hispanic OR AB=hispanic) OR (TI=latin* OR AB=latin*) OR (TI=indigenous OR AB=indigenous) OR (TI="native american" OR AB="native american") OR (TI=jewish OR AB=jewish) OR (TI=roma OR AB=roma) OR (TI="minority group*" OR AB="minority group*") OR (TI="ethnic and racial minorit*" OR AB="ethnic and racial minorit*”)))) AND ((TI=outcome OR AB=outcome) OR (TI=intervention OR AB=intervention) OR (TI=initiative OR AB=initiative) OR (TI=strateg* OR AB=strateg*)) AND ((TI="emergency medicine" OR AB="emergency medicine") OR (TI=EM OR AB=EM))	16 (14 duplicates were removed, resulting in two remaining articles)

Appendix B

Search terms for DEI outcomes and metrics used in PubMed, Scopus, and Web of Science, limiting results to English-language articles pertaining to the US from 2008 to the present, as presented in Table 5, yielded 5,073 results.

**Table 5:** Search for DEI metrics and outcomes (total n after deletion of duplications =5,073) DEI: Diversity, equity, and inclusion, MeSH: Medical subject headings

Database	MeSH terms	No. Of results
PubMed	1. ”Diversity, Equity, and Inclusion"[tiab] OR DEI[tiab] OR diversity[tiab] OR equity[tiab] OR inclusion[tiab] OR equality[tiab] OR equitab*[tiab] OR intersectionality[tiab] OR belonging[tiab] OR accessibility[tiab] OR inclusivity[tiab] OR "Diversity, Equity, Inclusion"[Mesh] OR "Cultural Diversity”[Mesh]; 2. compensat*[tiab] OR promot*[tiab] OR retention[tiab] OR "leadership representation"[tiab] OR hiring[tiab] OR "leadership opportunit*"[tiab] OR "pay equity"[tiab] OR turnover[tiab] OR "Personnel Turnover"[Mesh] OR "Achievement"[Mesh] OR "Salaries and Fringe Benefits”[Mesh]; 3. resident*[tiab] OR internship[tiab] OR physician*[tiab] OR fellow*[tiab] OR doctor*[tiab] OR surgeon*[tiab] OR allergist*[tiab] OR anesthesiologist*[tiab] OR cardiologist*[tiab] OR dermatologist*[tiab] OR endocrinologist*[tiab] OR gastroenterologist*[tiab] OR geriatrician*[tiab] OR gynecologist*[tiab] OR nephrologist*[tiab] OR hospitalist*[tiab] OR neurologist*[tiab] OR obstetrician*[tiab] OR oncologist*[tiab] OR opthalmologist*[tiab] OR otolaryngologist*[tiab] OR pediatrician*[tiab] OR physiatrist*[tiab] OR pulmonologist*[tiab] OR radiologist*[tiab] OR rheumatologist*[tiab] OR urologist*[tiab] OR "Physicians"[Mesh] OR "Education, Medical, Graduate"[Mesh]	3,962
Scopus	(TITLE-ABS("Diversity, Equity, and Inclusion") OR TITLE-ABS(DEI) OR TITLE-ABS(diversity) OR TITLE-ABS(equity) OR TITLE-ABS(inclusion) OR TITLE-ABS(equality) OR TITLE-ABS(equitab*) OR TITLE-ABS(intersectionality) OR TITLE-ABS(belonging) OR TITLE-ABS(accessibility) OR TITLE-ABS(inclusivity)) AND (TITLE-ABS(compensat*) OR TITLE-ABS(promot*) OR TITLE-ABS(retention) OR TITLE-ABS("leadership representation") OR TITLE-ABS(hiring) OR TITLE-ABS("leadership opportunit*") OR TITLE-ABS("pay equity") OR TITLE-ABS(turnover)) AND (TITLE-ABS(resident*) OR TITLE-ABS(internship) OR TITLE-ABS(physician*) OR TITLE-ABS(fellow*) OR TITLE-ABS(doctor*) OR TITLE-ABS(surgeon*) OR TITLE-ABS(allergist*) OR TITLE-ABS(anesthesiologist*) OR TITLE-ABS(cardiologist*) OR TITLE-ABS(dermatologist*) OR TITLE-ABS(endocrinologist*) OR TITLE-ABS(gastroenterologist*) OR TITLE-ABS(geriatrician*) OR TITLE-ABS(gynecologist*) OR TITLE-ABS(nephrologist*) OR TITLE-ABS(hospitalist*) OR TITLE-ABS(neurologist*) OR TITLE-ABS(obstetrician*) OR TITLE-ABS(oncologist*) OR TITLE-ABS(opthalmologist*) OR TITLE-ABS(otolaryngologist*) OR TITLE-ABS(pediatrician*) OR TITLE-ABS(physiatrist*) OR TITLE-ABS(pulmonologist*) OR TITLE-ABS(radiologist*) OR TITLE-ABS(rheumatologist*) OR TITLE-ABS(urologist*))	1,647 (987 duplicates were, resulting in 660 remaining articles)
Web of Science	((TI="Diversity, Equity, and Inclusion" OR AB="Diversity, Equity, and Inclusion") OR (TI=DEI OR AB=DEI) OR (TI=diversity OR AB=diversity) OR (TI=equity OR AB=equity) OR (TI=inclusion OR AB=inclusion) OR (TI=equality OR AB=equality) OR (TI=equitab* OR AB=equitab*) OR (TI=intersectionality OR AB=intersectionality) OR (TI=belonging OR AB=belonging) OR (TI=accessibility OR AB=accessibility) OR (TI=inclusivity OR AB=inclusivity)) AND ((TI=compensat* OR AB=compensat*) OR (TI=promot* OR AB=promot*) OR (TI=retention OR AB=retention) OR (TI="leadership representation" OR AB="leadership representation") OR (TI=hiring OR AB=hiring) OR (TI="leadership opportunit*" OR AB="leadership opportunit*") OR (TI="pay equity" OR AB="pay equity") OR (TI=turnover OR AB=turnover)) AND ((TI=resident* OR AB=resident*) OR (TI=internship OR AB=internship) OR (TI=physician* OR AB=physician*) OR (TI=fellow* OR AB=fellow*) OR (TI=doctor* OR AB=doctor*) OR (TI=surgeon* OR AB=surgeon*) OR (TI=allergist* OR AB=allergist*) OR (TI=anesthesiologist* OR AB=anesthesiologist*) OR (TI=cardiologist* OR AB=cardiologist*) OR (TI=dermatologist* OR AB=dermatologist*) OR (TI=endocrinologist* OR AB=endocrinologist*) OR (TI=gastroenterologist* OR AB=gastroenterologist*) OR (TI=geriatrician* OR AB=geriatrician*) OR (TI=gynecologist* OR AB=gynecologist*) OR (TI=nephrologist* OR AB=nephrologist*) OR (TI=hospitalist* OR AB=hospitalist*) OR (TI=neurologist* OR AB=neurologist*) OR (TI=obstetrician* OR AB=obstetrician*) OR (TI=oncologist* OR AB=oncologist*) OR (TI=opthalmologist* OR AB=opthalmologist*) OR (TI=otolaryngologist* OR AB=otolaryngologist*) OR (TI=pediatrician* OR AB=pediatrician*) OR (TI=physiatrist* OR AB=physiatrist*) OR (TI=pulmonologist* OR AB=pulmonologist*) OR (TI=radiologist* OR AB=radiologist*) OR (TI=rheumatologist* OR AB=rheumatologist*) OR (TI=urologist* OR AB=urologist*))	1,561 (1,110 duplicates were removed, resulting in 451 remaining articles)
